# Insight into Automatic Image Diagnosis of Ear Conditions Based on Optimized Deep Learning Approach

**DOI:** 10.1007/s10439-023-03422-8

**Published:** 2023-12-14

**Authors:** Heba M. Afify, Kamel K. Mohammed, Aboul Ella Hassanien

**Affiliations:** 1 Systems and Biomedical Engineering Department, Higher Institute of Engineering in Shorouk Academy, Al Shorouk City, Cairo Egypt; 2 Center for Virus Research and Studies, Al Azhar University, Cairo, Egypt; 3 College of Business Administration, Kuwait University, Kuwait, Kuwait; 4 Scientific Research Group in Egypt (SRGE), Cairo, Egypt; 5 Faculty of Computers and Information, Cairo University, Giza, Egypt

**Keywords:** Ear imagery database, Convolutional neural networks (CNNs), Hyperparameters, Bayesian optimization

## Abstract

Examining otoscopic images for ear diseases is necessary when the clinical diagnosis of ear diseases extracted from the knowledge of otolaryngologists is limited. Improved diagnosis approaches based on otoscopic image processing are urgently needed. Recently, convolutional neural networks (CNNs) have been carried out for medical diagnosis to obtain higher accuracy than standard machine learning algorithms and specialists' expertise. Therefore, the proposed approach involves using the Bayesian hyperparameter optimization with the CNN architecture for automatic diagnosis of ear imagery database including four classes: normal, myringosclerosis, earwax plug, and chronic otitis media (COM). The suggested approach was trained using 616 otoscopic images, and the performance of this approach was assessed using 264 testing images. In this paper, the performance of ear disease classification was compared in terms of accuracy, sensitivity, specificity, and positive predictive value (PPV). The results produced a classification accuracy of 98.10%, a sensitivity of 98.11%, a specificity of 99.36%, and a PPV of 98.10%. Finally, the suggested approach demonstrates how to locate optimal CNN hyperparameters for accurate diagnosis of ear diseases while taking time into account. As a result, the usefulness and dependability of the suggested approach will lead to the establishment of an automated tool for better categorization and prediction of different ear diseases.

## Introduction

Previously, biometric recognition systems used ear shapes which figured prominently for person identification in forensic, security, and building monitoring applications [1]. Currently, a challenge that becomes increasingly difficult when working with image recognition of various ear diseases to alleviate auditory loss [2]. In the context of otolaryngology, the otoscopic examination is a clinical ear test to assess its function and improve the diagnosis [3]. Accordingly, otoscopic images occupy a great niche in diagnosing diseases of the external and middle ear [4]. The common ear disease is called acute otitis media (AOM) that infects the middle ear, particularly in children [5]. There are some types of ear diseases including otitis media with effusion (OME), chronic otitis media with effusion (COME), chronic suppurative otitis media (CSOM), earwax plug, myringosclerosis, and serous otitis media (SOM). OME is defined as middle ear enlargement and fluid accumulation without bacterial infection. COME is defined as fluid staying in the middle ear and coming back without a bacterial infection that causes the hearing problem. CSOM is defined as a severe infection that has resisted conventional treatment and causes perforation of the eardrum. CSOM is one of the most prevalent infectious disorders affecting children. Earwax plug is defined as a buildup of earwax in the ear, which usually leads to hearing loss. Myringosclerosis is defined as a buildup of calcium in the eardrum without any symptoms. SOM is defined as a retracted and dull tympanic membrane. All these ear diseases are interrelated and may lead to serious developmental symptoms that are life-threatening [4]. The risk factors for ear diseases are based on the immune system, frequent colds, seasonal allergies, air pollution, gene mutation, and speech delays [6].

Based on WHO statistics [7] for ear disorders, nearly 2.5 billion patients suffer from different levels of ear disorders and at least 700 million will require hearing rehabilitation in 2050. It was noted that five out of six children develop otitis media by the time they reach the age of three. The good treatment of otitis media in children can be carried out by accurate diagnostic tools. Especially in developing countries, the inexperience of pediatricians leads to the wrong treatment and disability caused by ear diseases [8].

Portable otoscopy [9] is a standard test for ear inspection. This test is insufficient due to the resulting limited diagnostic accuracy. Besides, the common diagnostic tools for ear diseases mainly depend on head mirror, auriscope, otomicroscope, and video-endoscope [10]. An auriscope has a diagnostic accuracy than a head mirror, and it is insufficient to identify pathological structures of the ear. The otoscope is not suitable for a narrow ear canal due to limitations imposed by the size of the speculum. The best diagnostic tool is a portable otoscope with video-endoscope compared to other tools for determining the external ear canal, tympanic membrane, and normal or unusual constructions at these sites.

With the appearance of endoscopic ear surgery [11], many otolaryngologists began to use it in private clinics because of the high image quality and ideal illumination of this instrument to reach accurate inspection. However, the time and cost of this procedure are a disadvantage compared to manual otoscopy. From this point of view, early detection and appropriate treatment require new algorithms for ear image diagnosis to avoid the unavailability of otolaryngologists and low diagnostic accuracy at a lower time cost than the previous works [12]. In a clinical test, the correct diagnosis of OME achieved an accuracy of 43–52% [13], and the correct diagnosis of AOM achieved an accuracy of 62–78% [14].

Artificial intelligence screening systems for ear diseases could be used by healthcare workers in developing areas where there is a shortage of otolaryngologists. It could also reduce the number of clinicians by assigning a nurse to perform the first evaluation of individuals with ear complaints, which are exploited by automated diagnostic models. The automated diagnostic models based on machine and deep learning studies exist relatively in otology for the automated diagnosis of otoscope images as follows.

In terms of machine learning algorithms, Myburgh et al. [15] used an automatic system to diagnose five types of otitis media by applying it to a proprietary database including 389 ear images extracted from the video-endoscope. The diagnostic accuracy of this approach reached 81%-58% using a decision tree, and 86%-84% using the neural network method. Also, Vizcaino et al. [16] used a public database including 880 ear images by support vector machine (SVM) algorithm for classifying between four ear categories with an accuracy of 93.9%. Livingstone et al. [17] employed machine learning algorithms on 724 ear images with an accuracy of 89% using 14 classes. Sandström et al. [18] demonstrated good examination for ear diseases using machine learning algorithms on ear images detected by an expert panel.

In terms of deep learning algorithms, CNN models are applied to detect ear images as in Cha et al. [19] based on 10,544 private samples for six categories with an accuracy of 93.67%.

Zeng et al. [20] implemented 20,542 endoscopic images extracted from six categories to train nine CNN models with an accuracy of 95.59%.

Khan et al. [21] reported an accuracy of 95% using CNN models on 2,484 otoendoscopic images to distinguish them into three categories. Lee et al. [22] proposed the class activation map as feature extraction with CNN models on 1338 ear images to classify two types with an accuracy of 91 %. Zafer [23] proposed a combined approach extracted from CNN as feature extraction and machine model to classify 857 ear images with an accuracy of 99.47%. Moreover, this research suffered from a biased model and insufficient ear dataset. Recently, the classification of the public ear database used by Viscanio et al. [16] adopted the CNN-LSTM with good performance rather than CNN only [24]. This recent study confirmed that Bayesian optimization is a highly effective optimization technique because it is able to detect better hyperparameters in less time than other evolutionary techniques [24].

There are some limitations of previous works on ear diagnostic systems. It was observed that the number of ear images used in the machine learning algorithms was less than the images used in CNN models. As far as know, deep algorithms are more accurate for ear image detection due to their multilayered but have a high computational cost during model training [25]. Most ear detection systems are not executed in real-time diagnostics of the ear in clinics [20].

Therefore, the objective of this work is based on the optimized CNN approach based on four selected hyperparameters for providing an accurate otologic diagnosis with less time.

The primary feature of the propounded approach is applied to the available dataset which provides a large number of images used in [16] to classify four ear categories. Also, the proposed approach supported ear detection using CNN-based Bayesian optimization [26] as a supplement, but not as a replacement for the otoscope, which remains the typical screening tool among non-specialized physicians.

## Materials and Methods

### Proposed Framework

The basic target of the study is to include an accurate approach for classifying four ear conditions by Bayesian optimization [26] based on tuning the hyperparameters of a CNN architecture. Also, this proposed approach is used to minimize the training time of CNN without influencing the approach performance. This optimizer's advantage is its ability to identify the appropriate hyperparameters to obtain the best accuracy on the testing dataset.

**Fig. 1:**
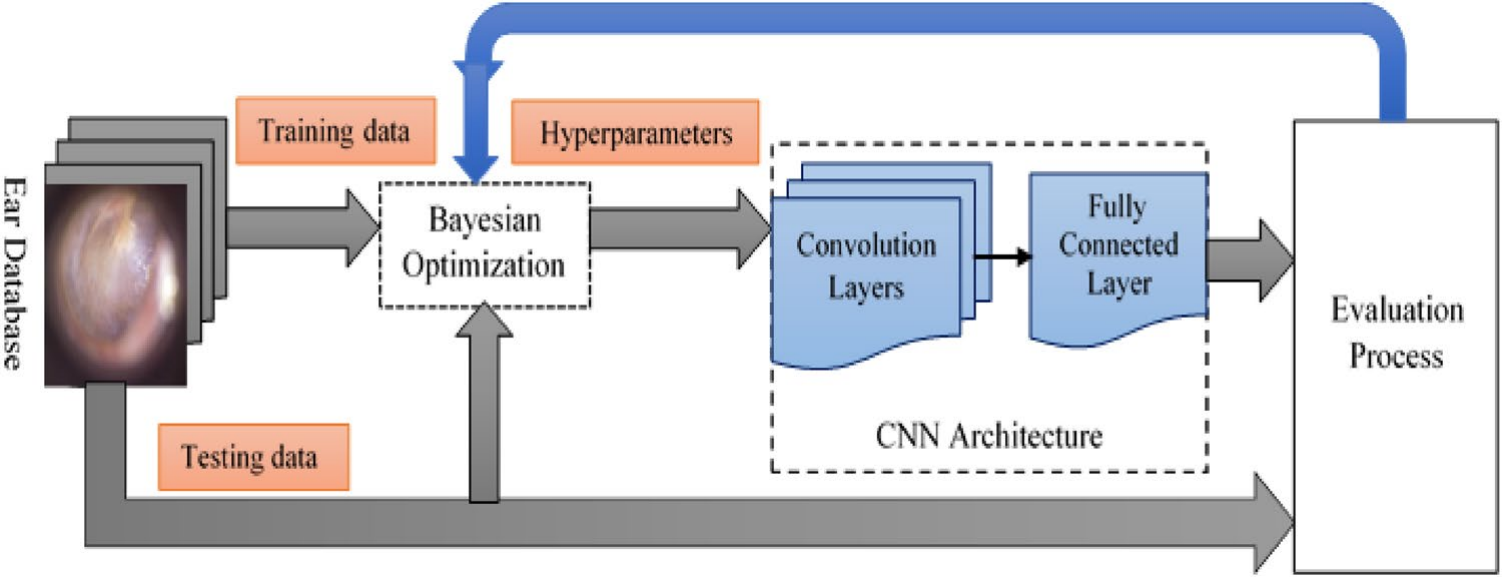
A proposed framework based on Bayesian optimization with the CNN for ear imagery classification

Figure 1 designs the proposed framework for the classification of ear diseases. The following contributions were adopted in the proposed approach.

1. Using Bayesian optimization can determine the optimal hyperparameter values in a shorter time.
2. Using the CNN architecture that contains convolutional layers followed by a fully connected layer to obtain a more accurate classification model.
3. Using the performance appraisal to compute the optimal indicators of 30 and 100 iterations. Also, a comparison with the previous methods is conducted.
4. Using the feedback structure to duplicate all aforementioned procedures for 30 and 100 iterations to determine the optimal outcomes.

### Database Acquisition

In this study, the ear imagery database [16] consists of 880 otoscopic images obtained from physicians in the Clinical Hospital of Universidad de Chile. These images were extracted from 180 patients and evaluated by an otolaryngologist according to four types including earwax plug, myringosclerosis, COM, and normal. Each type has 220 otoscopy images.

**Fig. 2:**
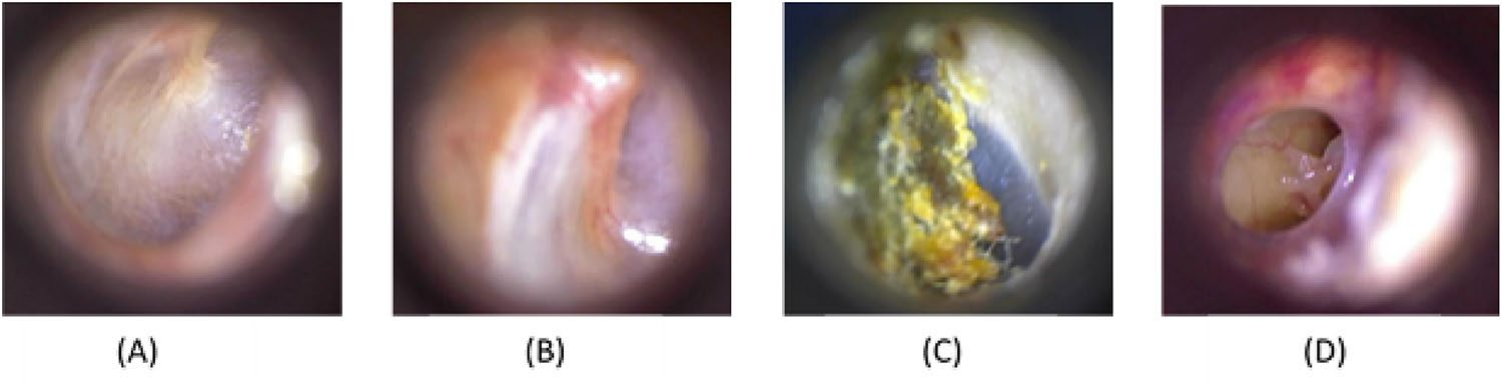
Otoscopy images for four diagnostic classes of ear diseases: **A** normal ear, **B** myringosclerosis, **C** earwax plug, **D** chronic otitis media (COM).

The otoscopic images are collected from 90 video images at 20 frames per second at a resolution of 640 x 480 for each case and the experts check the good frames identified by the jamming method from each of those video images. Then, it was found that the best-selected images were resized to 420 × 380 pixels. Fig.2 presents otoscopy images of four diagnostic classes of ear diseases in the ear image dataset.

In this paper, the database is indiscriminately partitioned into 70% for training data and 30% for testing data. It means that 880 images are divided into 616 training images and 264 testing images. For the implementation of the proposed approach, each class has 154 training images and 66 testing images. All images in the database were transformed to resized images with 32 × 32 pixels resolution for preprocessing data. The reason for resizing images to 32*32 is compatibility with proposed layers in deep learning methods, e.g., CNN. Also, resized images help in reducing the training time during the CNN method.

### Bayesian Optimization

To reduce computing costs and benefit from reliable CNN design, Bayesian optimization [27] is a potent technique for hyperparameter optimization. To choose the appropriate hyperparameters for the estimate process, this optimizer takes into account prior discoveries. When the data are complex, this optimizer is successfully employed to improve classification [28]. Hyperparameters are often a set of values employed in the learning process and are made up of an integer or variable that has values ranging from lower to higher bounds. Given the training duration, the best hyperparameters ought to have a low-loss function and excellent algorithmic accuracy. The selection of hyperparameters varies depending on the algorithm's goal.

The Bayes Theorem [29], which comprises prior knowledge of the goal function and updates posterior knowledge to reduce loss and improve classification precision, is a key component of the optimization process. The objective function's previous outcomes are updated using the posterior distribution, which is dependent on the Gaussian process [29]. Additionally, the acquisition function [26] is used to set a balance between exploring modern regions of the objective space and taking advantage of regions where suitable values are already known to exist. Depending on model Z and observation Y, Eq. (1) is Bayes' Theorem.

1 $$P\left(Z|Y\right)=(P\left(Y|Z\right)P\left(Z\right))/P(Y)$$

where P(Z|Y) is the posterior probability of Z given Y, P(Y|Z) is the likelihood of Y given Z, and P(Y) is the prior probability of Y.

The Bayesian hyperparameter optimization is generated by Eq. (2) as in previous work [26].

2 $$y=argmin f(y)$$

Y is a set of hyperparameters in the domain, and f(y) is an objective score to minimize the error rate in the learning process. The Bayesian optimization is constructed to locate the minimal function f(y) on a bounded set Y.

#### Hyperparameters

In this study, the selected hyperparameters are learning rate, momentum, regularization, and network depth as used in [26]. These four hyperparameters had previously produced good optimization results for images [31] as follows.

- Depending on the gradient loss function error, the learning rate (α) is utilized to identify the extensive patterns in images. Important patterns may inadvertently be left out if the learning rate is low.
- By updating the prior gradients, the momentum (δ) is used to adjust the entire image without losing significant components. To eliminate vertical oscillations and reroute a decent path to local optimums with lower iterations than the random gradient, the momentum value's goal is to facilitate the gradient descent processes.
- To get better forecasts without overfitting the database, regularization (λ) allows for good generalizability. To prevent the model complexity, the regularization is based on reducing the weight by a tiny amount known as weight decay. In this study, the loss function (L).-based computation of the squares of all the feature weights is employed to implement ridge regularization.
- The network depth is used to recognize the good features of the images by the accurate setting of the network.

The equations (Eqs. 3–5) for the hyperparameters are shown as the following:

3 $${\theta }_{new}\,=\,{\theta }_{old}-\alpha \left(\frac{\delta }{\delta {\theta }_{old}}\right)*gradient$$

The primary benefit of employing momentum is to smooth down the gradient descent steps, which reduces vertical oscillations and provides a smoother path to the local optimum with fewer iterations than the stochastic gradient. The gradient descent is made more efficient and produces better estimates by adding momentum [26].

4 $${V}_{t}={V}_{t}^{\delta }+1+\alpha \Delta wL(W,Z,y)$$

5 $$W=W-{V}_{t}$$

where Δw is defined as the gradient change, V_t_ is defined as the momentum variation according to the weight, and L (W, Z, y) is the loss function of weight (W), model (Z), and hyperparameters (y).

In Eq. (6), the loss function L is employed as an index for regularization (*λ*).

6 $$L(y,z)\,=\,\sum_{i=1}^{n}{(zi-yi)}^{2 }+\lambda \sum_{i-1}^{n}{\theta i}^{2}$$

where θi is the feature vector and y, z are the input parameters for the ith iteration.

In this paper, Table 1 displays the chosen hyperparameters for Bayesian optimization. Table 2 displays the ideal hyperparameters for five iterations using solely Bayesian optimization. The four chosen hyperparameters had an impact on the CNN computational time, as given in Table 3. Table 3 displays the best feasible locations for the hyperparameters tested with 30 and 100 iterations.

**Table 1:** Selected Bayesian hyperparameters for the proposed approach

Hyperparameters	Initial value	Final value	Type
Network depth	1	3	int
Learning rate (α)	1 × 10^-4^	1	log
Momentum (δ)	0.8	0.98	log
Regularization ( λ)	1 × 10^-10^	0.01	log

**Table 2:** Hyperparameter values for 5 iterations during the tuning process

Iterations	Learning rate (α)	Momentum (δ)	Regularization ( λ)	Network depth	Time (Seconds)
1	0.023901	0.9411	0.0013344	3	143.99
2	0.00014609	0.9627	0.00080658	1	98.713
3	0.051838	0.84494	1.7602 × 10^-9^	2	118.18
4	0.067281	0.80586	0.00012787	1	91.476
5	0.98837	0.81278	0.0027618	2	103.99

**Table 3:** Best hyperparameter values for 30 and 100 iterations

Iterations	Learning rate (α)	Momentum (δ)	Regularization ( λ)	Network depth
30 iterations	0.067281	0.80586	0.00012787	1
100 iterations	0.015771	0.80514	0.0093468	1

### CNN Architecture

The CNN architecture [32] successfully used medical image classification because it is based on the learning of features to achieve adequate realization without detailed information. For each iteration, the CNN consists of repeated three layers, including a convolutional layer, batch normalization layer, and a rectified linear unit (RELU) layer accompanied by a fully connected layer represented by the SoftMax classifier in the output layer.

In this paper, the max-pooling layer keeps the upper pixel values with its locative arranging to reduce the number of parameters over the training process. Hence, the function of a max-pooling layer is to gradually minimize the spatial size of the pattern to obtain fewer parameters and less time computation in the architecture.

### Bayesian Optimization for CNN Architecture

Some studies [33, 34] were performed to determine the amount of hyperparameters' influence on CNN design. There is a difference in the significance of the different hyperparameters. It has been observed that utilizing the incorrect hyperparameters increases both the time cost and classification error.

A good configuration of hyperparameters, such as learning rate, momentum, regularization, and network depth, was necessary for the CNN architecture to function well. It should be highlighted that the propounded network cannot be used to adjust the size and number of layers. The architecture and connectivity of the structure are taken into account as a sequential choice issue for this reason. The use of simple CNN architecture relied on tedious hyperparameter tuning and had a low degree of precision.

The optimizer's job is to use training samples to quickly identify the optimum hyperparameters. These techniques are addressed by the trial-and-error principle which is unsuccessful in selecting hyperparameters when compared to classic optimization methods [34]. Therefore, Bayesian optimization is appropriate for adjusting hyperparameters.

In this study, the fully connected layer and convolutional layers' hyperparameters for CNN architecture are tuned using the Bayesian optimization approach. The training and testing databases were inputs used by the Bayesian optimizer to generate the objective function. The objective function returned the classification error on the testing set after training a CNN architecture.

Four hyperparameters that were taken from the Bayesian optimization and training dataset were used as the input for the CNN architecture during implementation. The testing dataset and CNN architecture output are mixed to assess the classification process.

**Fig. 3:**
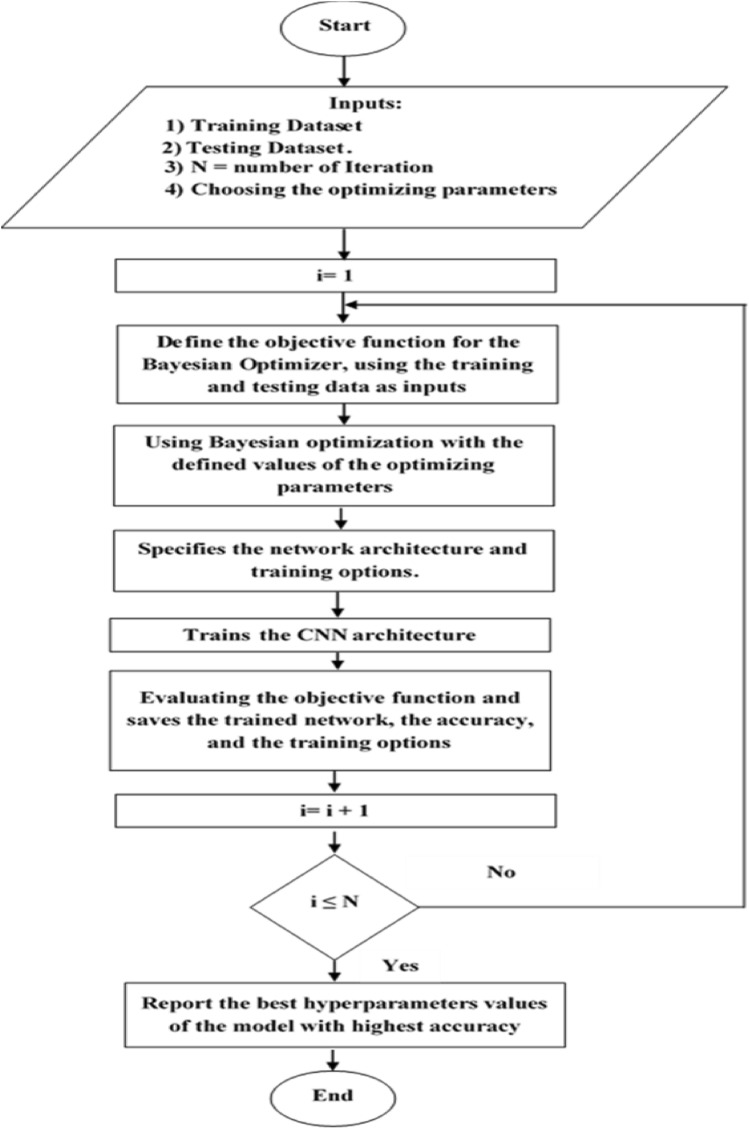
Flow chart of CNN-Bayesian optimization approach for ear imagery classification

As seen in Fig. 3, the following steps outline the Bayesian optimization of CNN architecture:

Step 1: Choose the initial hyperparameters for optimization from the images.

Step 2: Use an acquisition function to assess the objective function [30].

Step 3: Execute 30 and 100 iterations to determine the suitable iterations achieved in less time.

Step 4: Choose the most optimal values.

Step 5: Make use of the test database's optimized hyperparameters.

### Optimization Setup

In this paper, the implementation of the proposed approach is performed on a laptop with 5 GB RAM GPU under the MATLAB 2020 program. The experiments are evaluated on otoscopy images for classifying ear diseases acquired from [16]. This work contains a total of 880 otoscopy images. To assess the model performance, the ear disease detection approach is employed on these images by using four common criteria: accuracy, sensitivity, specificity, and PPV, as used in former work [16]. Also, the receiver operating characteristic (ROC) curve is widely applied to compare the model performance in various classifiers [36].

The overall efficiency of a classifier is its accuracy. Specificity is used to correctly identify the samples free of illnesses, whereas sensitivity is utilized to prevent false negative samples. To determine the accurately categorized samples based on the total number of classified samples, PPV is employed as a precision parameter. For each criterion, the best performance is represented by the maximum of these percentages [37].

## Experimental Results

The limited confidence during otoscopy examination to detect ear is adequate to warrant a new strategy for diagnosing various ear diseases [38]. Considering some previous studies regarding the diagnosis of ear diseases, the automated diagnosis based on otoscopy image classification models can perform better than otolaryngologists, as this classification contributes to the early diagnosis of ear diseases, especially for clinical suspicion. Interestingly, this proposed approach suggested that automatic ear detection by CNN models should be an addition to, rather than a replacement for, otoscopes in ear disease diagnostic methods.

In this research, a total of 880 otoscopy images were applied to train two CNN models and CNN-Bayesian optimization by four hyperparameters to select the best model. The performance of three architectures on the ear imagery dataset containing normal, myringosclerosis, earwax plug, and COM is evaluated in terms of four criteria [37].

### Performance Analysis

In the first stage, the two CNN frameworks used DarkNet-19 [39] and inception-v3 [40] on the four ear diseases for the classification task. Inception-v3 [40] and DarkNet-19 [39] were originally designed for different applications, and these architectures are fundamentally sound for image classification tasks. In our study, we chose to use Inception-v3 and DarkNet-19 as reference classifiers due to their well-established performance in image classification tasks, although they have no application to ear disease diagnosis.

In the second stage, the CNN framework employed Bayesian optimization using four hyperparameters, including learning rate, momentum, regularization, and network depth, during the development and testing of the suggested methodology for the autonomous diagnosis of four ear disorders. The layers are used to quickly optimize the hyperparameters, and the testing dataset is used for extensive optimization.

In this study, the CNN framework was exploited to recognize the four groups obtained from the ear imagery dataset [16] which every image has a dimension of 32 × 32. The total ear database occupied 880 images which have 616 images for training and 264 images for testing. We tested the proposed framework with 264 test images that were not used in the training process for the diagnosis task. The sum of convolutional layers is 3 stacks. Each stack consists of a convolutional layer, batch normalization layer, and RELU layer. The image volume is 32 × 32 × 3, where 3 indicates the image depth. The minimum batch size is 128. The first number of filters is round (16/√ (Ov × network depth)), where Ov is the optimizing value applied to retain the same training values. The filter size along layers is 32 × 32, 16 × 16, and 8 × 8, respectively.

According to Table 3, the CNN-Bayesian optimization approach is applied to the best values of hyperparameters for two iterations including 30 and 100. To reduce the generalization error and improve classification accuracy, the optimal hyperparameter values are used for iterations. Using the testing set (30%), the acquired results for the CNN-Bayesian optimization approach were reported accuracy of 98.10%, sensitivity of 98.11%, specificity of 99.36%, and PPV of 98.10%, for 30 iterations as shown in Table 4.

**Table 4:** Performance analysis of the CNN-Bayesian optimization approach, Darknet-19, and Inception-v3 on the 264 testing images extracted from the ear imagery database

	Accuracy %	Sensitivity%	Specificity%	Positive predictive value (PPV) %
CNN-Bayesian optimization	98.10	98.11	99.36	98.10
Darknet-19 [39]	34.09	34.09	78.03	31.96
Inception-v3 [40]	87.50	87.50	95.83	87.76
SVM [16]	93.90	87.80	95.90	87.70

On the other hand, the classification tests using the validation set (10%) and testing set (20%) showed that the CNN-Bayesian optimization approach reported an accuracy of 96.5%, sensitivity of 96.5%, and specificity of 98.8% for 30 iterations. Moreover, the results showed that the CNN-Bayesian optimization approach using training and testing sets was superior to the CNN-Bayesian optimization approach using a validation set.

The confusion matrix represented the classifier's accuracy tool that depended on the relationship between the correctly predicted images in the diagonal of the matrix and the incorrectly categorized images outside of the diagonal.

**Fig. 4:**
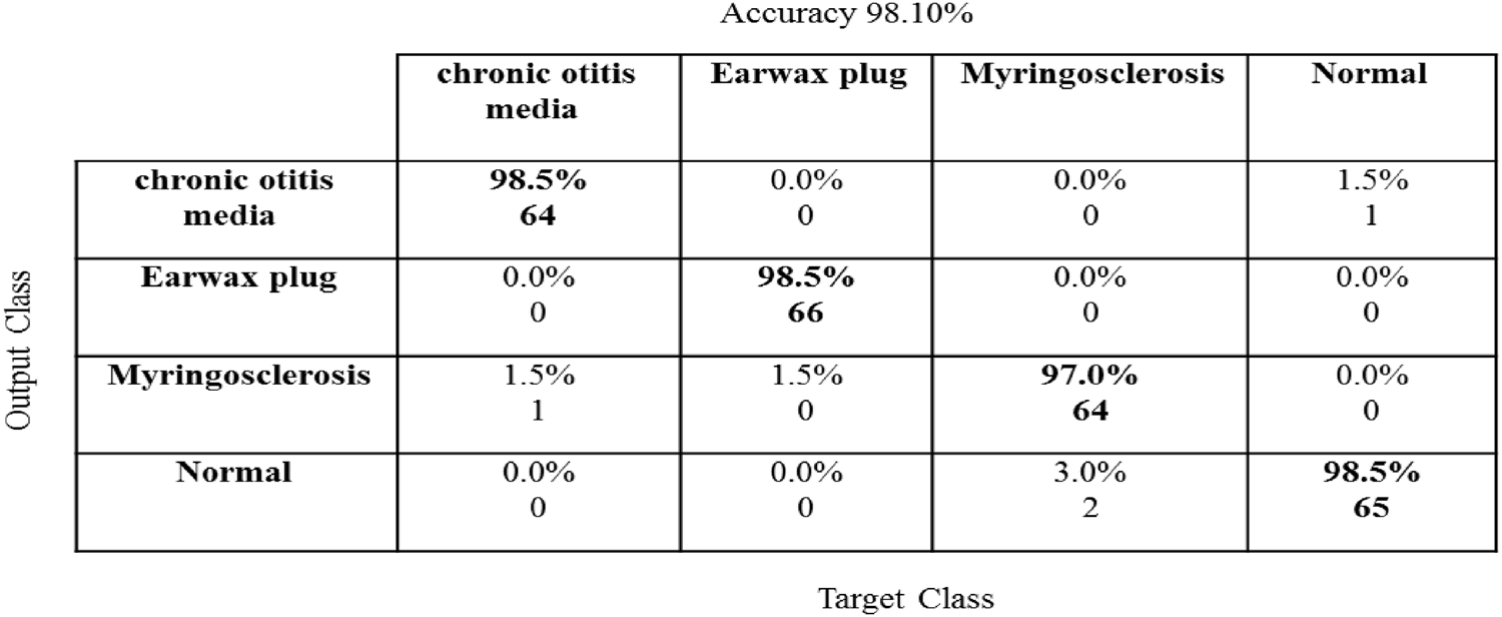
Confusion matrix for four ear classes using CNN-Bayesian optimization with 264 testing images

In this paper, a confusion matrix for four ear categories using CNN-Bayesian optimization with 264 testing images is superior to the previous works, as shown in Fig. 4.

**Fig. 5:**
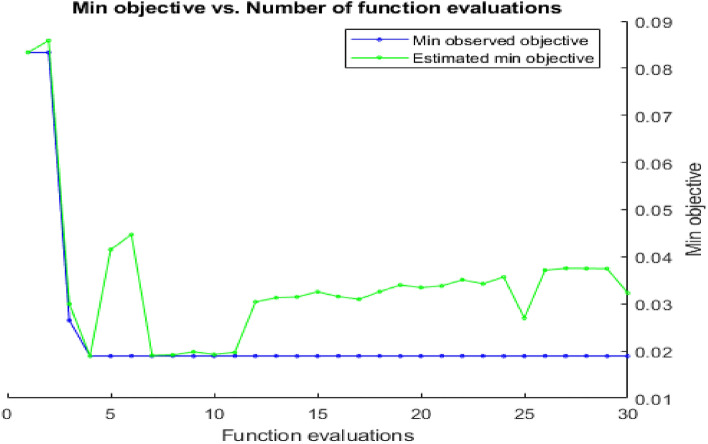
Minimum objective and number of function evaluations by using CNN- Bayesian optimization for 30 iterations

**Fig. 6:**
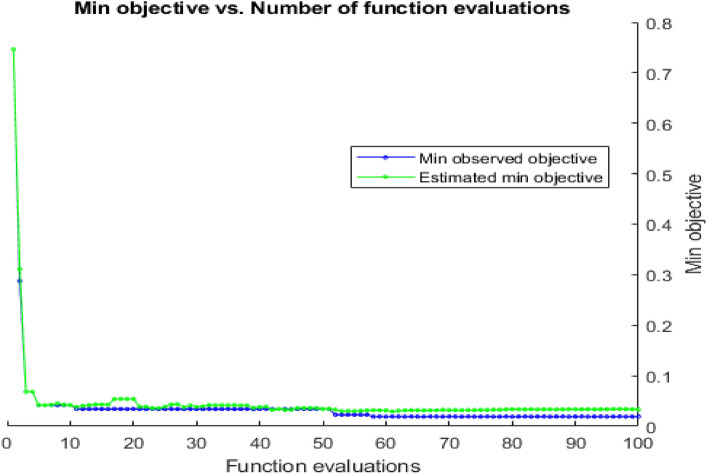
Minimum objective and number of function evaluations by using CNN-Bayesian optimization for 100 iterations

The images taken from the acquisition function are approximated over the objective function for each iteration. Through a feedback system, the images are introduced to the data to refresh its posterior. The posterior distribution is motivated by the objective function using a Gaussian process. By using CNN-Bayesian optimization. Figure 5 demonstrates the relationship between the minimal target attained after 30 iterations and the quantity of function evaluations. Figure 6 depicts the relationship between the minimal target attained after 100 iterations and the quantity of function evaluations.

### Training Time Analysis

The training time for detecting the ear imagery dataset for 30 iterations is 3136.9 seconds and the time of objective function evaluation is 3138.7 seconds, as shown in Table 5. On the other hand, the training time for detecting the ear imagery dataset for 100 iterations is 5608.2 seconds and the time of objective function evaluation is 5374.9 seconds, as shown in Table 5. It means that the CNN-Bayesian optimization approach with 30 iterations achieved a lower training time for the detection of the ear imagery dataset when compared to the proposed approach with 100 iterations.

**Table 5:** Training time of the CNN-Bayesian optimization approach for the detection of ear imagery dataset using 30 and 100 iterations

Iterations	Total elapsed time (s)	Total objective function evaluation time (s)
30 iterations	3163.9	3138.7
100 iterations	5608.2	5374.9

**Fig. 7:**
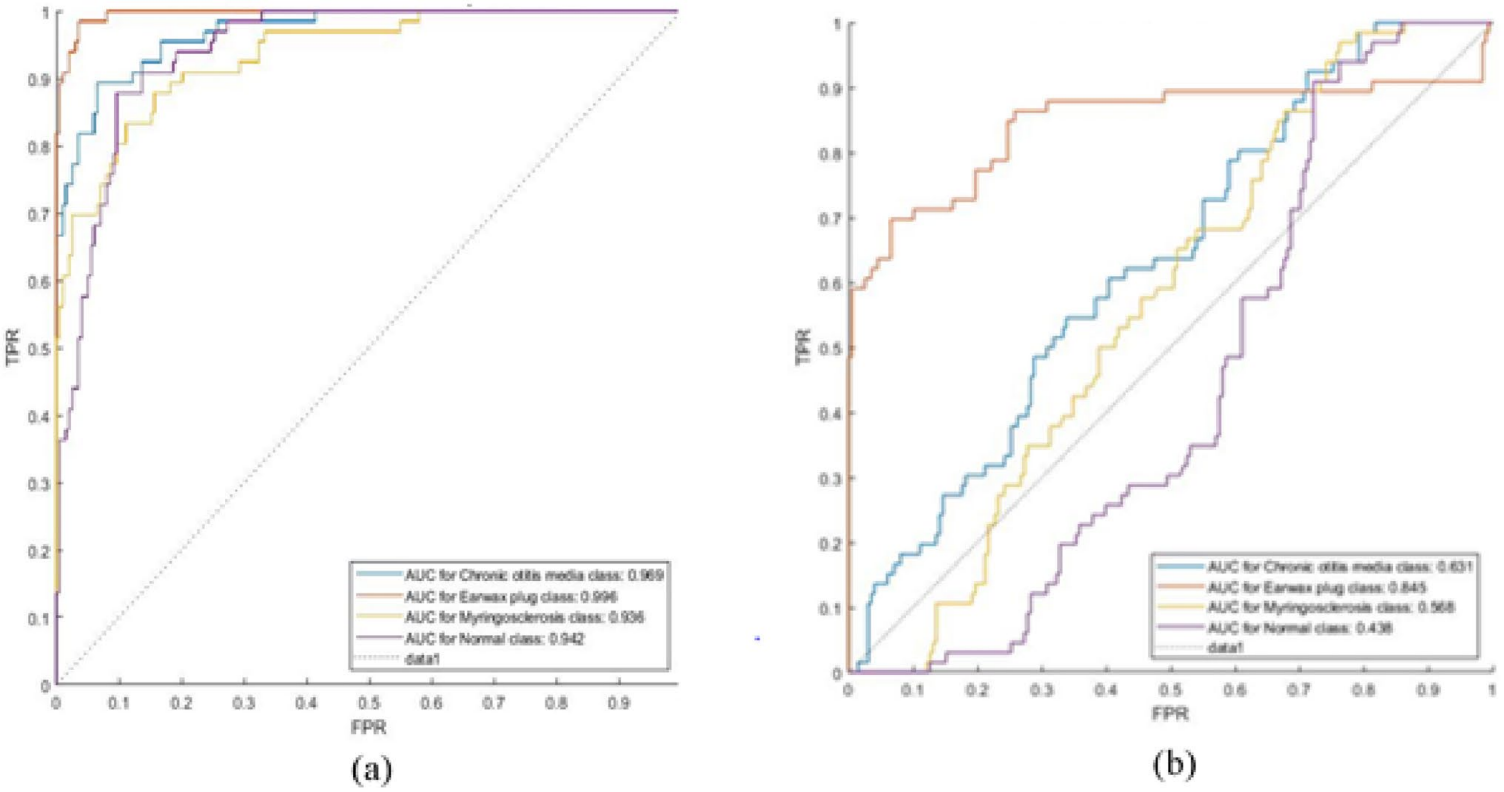
ROC Curve for ear diseases classification using two approaches **a** Inception-v3, **b** Darknet-19

In this paper, two CNN approaches (DarkNet-19 [39] and inception-v3 [40]) are used and compared the performance between the proposed approach and these two approaches on the same database [16]. The appropriate parameters for Darknet-19 and inception-v3 applied on the ear imagery database for one iteration are presented in Table 6. The ROC curves [36] for ear imagery classification using Darknet-19 and inception-v3 approaches are illustrated in Fig.7. It means that the inception-v3 approach achieved a higher result than the Darknet-19 approach as illustrated in Table 4. As inferred from Table 4, the experimental findings confirmed that this proposed approach is more accurate and effective for ear condition classification than CNN's previous works [39, 40]. For one iteration, the training time on Darknet-19, Inception-v3, and CNN-Bayesian optimization networks is 5580 seconds, 44880 seconds, and 80 seconds, respectively. After investigating three different networks, our results showed that the CNN-Bayesian optimization is the optimal network for detecting four imaging patterns of ear diseases with less training time for one iteration, as shown in Table 7. Therefore, using the CNN approach only is not efficient for the accurate diagnosis of ear diseases. Also, it was observed that ear detection by CNN models required a longer time.

**Table 6:** Selected parameters for Darknet-19 and inception-v3 approaches applied to the ear imagery database

	Momentum	Learning rate	Regularization	Mini. Batch Size	Max. Epochs
Darknet-19 [39]	0.9	1e−4	1e−4	15	6
Inception-v3 [40]	0.4	1e−5	1e−4	25	100

**Table 7:** Training time of the CNN-Bayesian optimization approach and two CNN's previous works on the same database

	Total time for one iteration (s)
CNN-Bayesian optimization	80
Darknet-19 [39]	5580
Inception-v3 [40]	44880

## Discussion

Instead, an otoscopy image is merely a screening tool for ear pathologies. This study focused on a second opinion for estimating the diagnostic performance of otoscope images [41].

The propounded approach helps in diagnosing ear diseases mainly depends on otoscopy images and physicians’ practice. For otology, ear disease detection by otolaryngologists is not easy [42].

Many researchers adopted both accessible and inaccessible databases of image detection techniques for ear diseases. Due to the variations in the samples, classes, and applied procedures, it was highlighted that there is no way to effectively compare earlier works of ear disease diagnosis. The significance of this work is to achieve the rapid and accurate classification of images of ear ailments such as normal, myringosclerosis, earwax plug, and COM.

The suggested approach took advantage of a modern public database, specifically the dataset of 880 ear images [16] that had previously been implemented to machine learning algorithms with an accuracy of 93.9%, a sensitivity of 87.8, a specificity of 95.9%, and a PPV of 87.7%.

Besides, the private dataset of 389 images was used with 86.8% accuracy by machine learning algorithms [15]. This indicates that the dataset for the ear with big images is more useful for enhancing the classification procedure. The lack of large sample sizes in the otolaryngology sector imposed numerous limitations on CNN design.

Among the three multi-classification models, CNN-Bayesian optimization achieved higher yield estimation accuracy relative to Inception-v3 and Darknet-19 models. In the proposed research, the optimized CNN architecture is applied to differentiate ear classes in the ear imagery dataset and obtained an accuracy of 98.10%, a sensitivity of 98.11%, a specificity of 99.36%, and a PPV of 98.10%. When the suggested approach was tested, the test error rate was 1.9%, indicating a reduced rate. These results performed better than what is stated in the literature [16]. Once it had reached the maximum optimized values, the Bayesian optimization automatically halted.

The Bayesian optimization for CNN architecture produced 616 training images (154 per class) to create the optimal hyperparameters that support the multi-class classification procedure. The multi-class classification procedure produced 264 testing images (66 per class) to track how well the approach performed according to the four metrics that were considered. We notice that the best results of the proposed approach were achieved by performing a classification stage without a validation set, using the training and testing sets.

The comparison between the Inception-v3, Darknet-19, and CNN-Bayesian optimization approach concerning four criteria is shown in Table 4. Also, it was found that the performance of the proposed approach for 30 iterations was the same as for the proposed approach for 100 iterations. However, the proposed approach for 30 iterations could be a useful time-saving tool for screening ear diseases, and this is especially true in areas where there is a shortage of otolaryngologists. From experimental outcomes, the combination of CNN with Bayesian optimization is an effective tool in ear diagnosis where human experience is inadequate to improve classification accuracy. The limitation of this study depends on a recent ear database and fewer studies dealing with ear image databases.

Furthermore, this is the first survey to use a CNN schema with Bayesian hyperparameter optimization to classify otoscopy images into four diagnostic categories using a total of 880 images. This study is a good choice whether the otoscopy images were taken by otolaryngologists or non-otolaryngologists. The main issues handled in this study are minimizing the dependence on large training images and reducing the time when the CNN is used.

Considering some limitations of the current study, this proposed approach did not consider external factors for patients with ear complaints such as age, fever, ear fullness, and environmental changes in the diagnostic process. There is no correlation between clinical information and the current classifier's results based on otoscopy images.

Regarding the implications of the obtained results, the current study suggested the demand for a sufficient database for ear diseases. In future, the accurate diagnostic coverage in the proposed approach will be beneficial for physicians with less experience; thus, it may reduce the growing number of hearing-impaired patients.

## Conclusion and Future Trends

In the era of ear disorders, it is difficult for an otolaryngologist to reach a precise diagnosis, which can have negative implications for treatment decisions. This field has been plagued by issues in otologic diagnosis related to limited consciousness, false detection, and a paucity of ear databases. This proposed approach could address these issues with CNN based on Bayesian hyperparameter optimization to provide an automatic diagnosis for patients with ear diseases who have otoscopic images. It was observed that the proposed approach is excellent analytical for multi-classification through four ear diseases. Ultimately, otology image processing will improve otolaryngology patient care, and ongoing work will boost the understanding of prediction models for rare specialized illnesses.

## Data Availability

Data are available upon request.
